# AI as a Therapist, Companion, and Romantic Partner: Emerging Roles, Benefits, and Risks for Mental Health in Participatory Medicine

**DOI:** 10.2196/95153

**Published:** 2026-08-05

**Authors:** John Grohol

**Affiliations:** 1 Society for Participatory Medicine Newton Highlands, MA United States

**Keywords:** artificial intelligence, AI, chatbots, large language models, digital mental health, participatory medicine, human-AI interaction, loneliness, AI companionship, romantic AI, mental health technology, patient empowerment, shared decision-making, online disinhibition

## Abstract

The line between tool and companion was once obvious, but conversational AI is blurring it in ways few researchers anticipated. Large language model chatbots and purpose-built AI companion agents are now used by millions of people every day. They are not being used to simply retrieve information but, instead, to offer emotional support, help process personal distress, and sustain what many describe as genuine relationships. Research puts the scale of this shift in sharp relief as nearly half (48.7%) of individuals with self-reported mental health concerns report having used a large language model for mental health support or therapy-related purposes. This Viewpoint argues that these uses are best understood through 3 unique but overlapping relational frames: AI as a therapist substitute, AI as a companion or confidant substitute, and AI as a romantic partner substitute. Drawing on empirical literature across digital mental health, psychology, communication, and human-computer interaction and grounded in the values of participatory medicine, this paper examines why people turn to AI for these intimate purposes; what they appear to gain; and what clinicians, designers, developers, and policymakers should examine more carefully as the practice evolves. The picture that emerges is neither straightforwardly optimistic nor dismissive. Therapeutic chatbots can produce real symptom reduction for users; AI companionship can ease loneliness in genuine, if bounded, ways; and the emotional relief some people experience in these interactions is not an artifact of naivety. But the same systems that lower the barriers to disclosure also lower the barriers to harm. AI chatbots regularly hallucinate clinical guidance, validate dysfunctional beliefs, handle crises without accountability, and may cultivate the very isolation they seek to relieve. Responsible integration requires something more demanding than a disclaimer. Instead, it requires transparent design, thoughtful escalation pathways, ongoing evaluation, and a commitment to the human connection that participatory medicine places at the center of good care.

## Introduction

Nearly half of people (48.7%) who have mental health concerns have turned to a large language model (LLM) for emotional support and guidance [1]. This is a significant shift in mental health technology use, notable for its rapid adoption and unique relational character. People are not using these systems merely as symptom trackers or medication reminders but also as psychological helpers, coaches, friends, and, in some cases, intimate partners. Understanding this shift requires an objective look at both what these systems can and cannot offer.

Historically, digital mental health tools have been largely designed to be structured and bounded. Web-based cognitive behavioral therapy (CBT) programs, symptom monitoring applications, and clinician-supported telehealth platforms were built around defined protocols with clear boundaries between tool and clinician. Research suggests that these interventions can improve reach and scalability but also acknowledges that the data, implementation quality, and regulations remain uneven [2]. Contemporary LLM-based systems represent a qualitative departure from this paradigm. They engage in open-ended natural language dialogue, respond dynamically to whatever the user brings, and can remember context within and across sessions. General LLMs do not follow a protocol but, instead, follow the conversation wherever it may lead. This responsiveness is what makes them feel relational, making a person feel connected to the AI agent, but it can also make evaluating them in research challenging.

The relational patterns that have emerged can be organized around 3 overlapping but conceptually unique roles. First, individuals are using AI as a therapist substitute. They use it to describe psychological distress, request coping strategies, work through emotional issues, and sometimes conduct extended quasi-therapeutic dialogues over weeks or months. Second, users can relate to AI as a friend or companion. They turn to it for social contact and validation, the simple relief of feeling heard, and the comfort of full-time availability that human relationships cannot guarantee. Third, users pursue AI as a romantic partner. They use it to seek affection, intimacy, and the experience of being desired in ways that can feel more accessible than in real-life connections. These categories are ideal types, but real use is messier and moves between them. However, the distinctions matter because the risks meaningfully differ and because the clinical and ethical obligations they generate are not identical.

What makes AI especially suitable for these roles are several key features. These features include their nonjudgmental attitude, 24/7/365 availability, the absence of social costs that normally regulate disclosure, and their continuity over time. Communications research has documented the human tendency to respond to computers as social actors, applying interpersonal norms to systems even when we know them to be nonhuman [3]. Conversational AI intensifies this tendency by adding responsiveness, attunement, and what can feel like genuine interest. Research demonstrates that people disclose more sensitive information to computer agents than to human interviewers because the felt absence of social evaluation lowers the stakes of disclosure [4]. In mental health contexts, that lowered threshold is both an asset and a vulnerability.

The limitations of these systems are primarily structural. Most general LLM chatbots are probability systems trained to produce plausible and engaging language. They are not trained to reason clinically, and they have no mechanism for checking the accuracy of what they say. The extensive literature on hallucination in natural language generation makes clear that fluency and correctness are largely independent properties. That is, a model can produce confident, well-structured, and completely fabricated clinical guidance with equanimity [5].

A second limitation concerns how AI engagement systems are generally designed. Psychotherapy can be seen as a practice of clarification, explanation, and productive disruption. A clinician gently challenges a person’s distorted thoughts (or *cognitions*), asks them to tolerate the resulting discomfort, helps them understand and replace the distorted cognition, and holds the space throughout the process. AI systems are optimized for engagement and, therefore, are structurally incentivized to do the opposite: agree, affirm, and keep the conversation going. Sycophancy research on language models confirms this tendency. LLMs preferentially validate the user’s expressed views, particularly when the user signals high confidence or investment in those views [6]. In a therapeutic frame and relationship, this is a direct contradiction of one of therapy’s core functions.

This Viewpoint engages these themes through the lens of participatory medicine, which holds that good health care is fundamentally relational. It is built on active patient engagement, shared decision-making, and partnership between clinician and patient that treats the patient as a knowledgeable, autonomous agent rather than a passive recipient of expertise. Conversational AI can advance some of these values by democratizing access to information and reflection. However, participatory medicine also wants tools to be honest about what they are, support rather than supplant human judgment, and strengthen rather than erode the social fabric through which real health is sustained.

## AI as Therapist

The mental health workforce crisis is worsening. Across most high-income countries, the gap between the prevalence of mental illness and the availability of treatment is widening. Worldwide, the median stands at just 13 mental health workers per 100,000 population, and while high-income countries report more than 60 workers, approximately half the world’s population lives in countries with only 1 psychiatrist for every 200,000 or more people [7]. In the United States, federal workforce modeling projects a shortage of roughly 43,800 psychiatrists and nearly 99,800 psychologists by 2038 and notes that these figures reflect only current service use—accounting for unmet need pushes the projected gaps substantially higher [8]. This gap represents a structural failure that leaves most of those who need care without adequate access. The World Health Organization’s World Mental Health survey found that between 35.5% and 50.3% of serious cases in high-income countries received no treatment in the preceding 12 months, with far worse figures in settings with fewer resources [9]. Even in high-income countries, only about one-third of people with major depressive disorder receive formal mental health care, and among those who are treated, just 23% receive care that meets the minimum standards of adequacy [7]. Mental health worker shortages exist throughout the United States, with nearly 50% of citizens living in shortage areas [10]. Wait times extend the problem even for those who do seek care. A national study of US psychiatry practices found that only 18.5% of psychiatrists could see new patients, with a median wait time of 67 days for in-person appointments [11]. Geography further limits options. An estimated 65% of nonmetropolitan US counties have no practicing psychiatrists, leaving over 60% of rural Americans in designated mental health care provider shortage areas [12]. High treatment costs can make access difficult even where services nominally exist. In addition, stigma suppresses help-seeking behaviors well before other barriers come into play [13]. Against this backdrop, the appeal of a system that is available at any time, with minimal or no cost, and does not ask intrusive questions is not difficult to understand. Novel models for reducing the burden of mental illness at a population scale have been an active research priority for more than a decade [14], and digital tools are regularly discussed as one component of any realistic solution [2].

The case for AI-assisted mental health support is more than theoretical as there are many randomized controlled trials supporting its use. A randomized controlled trial found significant reductions in depressive symptoms among young adults assigned to the Woebot conversational agent compared with an information-only control group, with positive effects emerging after just 2 weeks [15]. A systematic review and meta-analysis of mental health chatbots found small to moderate improvements in depression and anxiety scores across studies [16]. Research limitations of these studies include weak or nonexistent comparison groups, short follow-up periods, and lack of longitudinal data. Because of these limitations, the data do not conclusively establish that chatbots are equivalent to psychotherapy. However, under certain conditions using purpose-built AI chatbots, the research suggests that they can produce clinically significant outcomes.

The therapeutic benefits of these chatbots seem to occur for 2 different reasons. The first is greater disclosure. A study by Lucas et al [4] found that participants assigned to interact with a computer agent rather than a human interviewer reported more sensitive information, including depression and posttraumatic stress disorder symptoms, with measurably lower concealment behaviors. Participants knew that they were talking to a machine, so this finding cannot be explained by technical naivety. Rather, the social costs of disclosure—such as the fear of judgment, embarrassment, or unwanted consequences—are simply lower when the audience is perceived as nonhuman. For individuals whose mental health concerns carry significant stigma [13], this reduction in social cost may help increase disclosure. The second mechanism is structured reflection. Many purpose-built mental health chatbots incorporate CBT-derived techniques that can be meaningfully facilitated through conversational prompting without the need for a human therapist [15,16].

Challenges arise when users shift from purpose-built tools to general-purpose LLMs. The risks are categorically different. Structured therapeutic chatbots generally have a defined scope, documented safety protocols, relational guardrails, and clinical oversight built into their design. In contrast, a general LLM has none of these components. It can generate confident-sounding clinical guidance, medication recommendations, diagnostic interpretations, and therapeutic advice but lacks a mechanism for distinguishing between what it knows and what it has fabricated. The research literature on hallucinations makes it clear that language fluency is not the same as truth and modern generative systems can produce fabricated facts, invented references, and mischaracterized evidence in the same tone and confidence they use when conveying accurate information [5]. In mental health contexts, where users may be making decisions about medication, symptom interpretation, or crisis escalation, there may be significant consequences of providing confident misinformation.

Sycophantic alignment compounds this risk in ways that are harder to detect. A hallucination can be fact-checked, but sycophantic distortion cannot. General LLMs tend to align with whatever the user wants to hear, validating their beliefs, confirming their suspicions, and reinforcing their framings rather than offering the gentle friction that characterizes effective therapeutic dialogue. Research documents that this effect is strongest when the user signals confidence or emotional investment [6]. This is exactly the condition that most frequently accompanies entrenched maladaptive cognitions. A chatbot that confirms a person’s worst fears or validates false beliefs is not failing by the metrics it has been optimized for. It can be maximizing engagement while systematically worsening the user’s state of mental health and well-being.

User crises expose a further limitation. A therapist’s duty of care extends beyond a single psychotherapy session through ongoing risk assessment, legally obligated reporting, safety planning, and the capacity to escalate when a patient needs more help. General conversational AI lacks these capabilities. It cannot verify follow-through on a safety plan, contact emergency services, or assume legal accountability for the advice it provides. The gap between the promise of accessible mental health support and the practice of clinically responsible care is at its widest here. For users in crisis, AI is not a substitute for human intervention regardless of how sophisticated its responses become [17].

## AI as Friend or Companion

Loneliness is one of the most consequential public health problems of our time, associated with elevated all-cause mortality, accelerated cognitive decline, worse cardiovascular and immune outcomes, and substantially elevated risk of depression and anxiety [18]. A meta-analysis of 70 studies by Holt-Lunstad et al [18] found that social isolation, loneliness, and living alone each independently predicted a 26% to 32% increased likelihood of premature mortality, effects comparable in magnitude to those of well-established behavioral risk factors.

The health care system has very few tools to address this issue. It can be diagnosed, patients can be counseled, and it can be noted as a risk factor, but clinicians cannot prescribe social connection. Conversational AI has arrived with the promise of helping alleviate loneliness, and emerging research suggests that it can indeed sometimes be helpful.

Studies on human-chatbot relationships demonstrate what users experience. However, this research tends to be more subjective and based on behavioral observations rather than the randomized controlled studies used to examine AI use in therapy. Skjuve et al [19] followed users of a social chatbot over time and found something resembling genuine relationship development. Their research found relationships that involved increasing trust, escalating self-disclosure, a sense of being known and responded to individually, and positive increases in a person’s well-being [19]. Participants were not confused about whether they were talking to a machine, and they reported that the interaction provided something of value. A study by Merrill et al [20] found that social presence, the sense that one’s friend is meaningfully “there” and present, and perceived warmth were the key mediating variables through which AI companion interactions affected loneliness and willingness to engage. This makes sense because loneliness is fundamentally not about the absence of physical company so much as it is about the absence of felt responsiveness. Loneliness is the experience of being unseen and unheard.

Additional studies support AI’s use to help reduce feelings of loneliness. A number of studies of social chatbot use suggest that users may perceive these systems as friends and sources of emotional support, particularly when interactions feel responsive, nonjudgmental, and consistently available [21,22]. De Freitas et al [23] report significant loneliness reductions associated with AI companion use, with effects consistent across multiple studies. Limitations associated with this research include selection effects, where lonely people are more likely to adopt the use of AI chatbots, and various confounds. In addition, the long-term effects of companionship agents remain poorly understood.

The features that make AI a reliable conversational partner—consistent availability, patience, and unfailing attentiveness—simulate qualities that are scarce in human relationships precisely because they are challenging to provide. When an AI companion is described as “always there,” this is noting an asymmetry not typically found in human relationships. Research explains why this asymmetry is not psychologically salient in the moment [3]: the mind responds to socially cued stimuli with social processing regardless of its knowledge of their origin. However, the asymmetry is real, and it carries risks that imperceptibly emerge over time rather than in any single interaction.

According to the social displacement hypothesis, time and emotional investment in AI companionship may come at the cost of investment in human relationships. The review by Nowland et al [24] on loneliness and social internet use finds a complex interaction. Digital interaction can facilitate reconnection and provide a bridge back to human community. However, the researchers also found that, for some individuals, particularly those who already find human interaction aversive or threatening, digital substitution becomes an avoidance strategy that deepens isolation over time [24]. The distinction matters for participatory medicine because the appropriate clinical response will differ for someone using AI companionship as a bridge vs someone using it as a retreat.

There is also a privacy dimension to companionship use that is easy to forget. People tell their AI companions things that they have not told their clinicians, partners, or closest friends, such as trauma histories, relationship grievances, health anxieties, or even expressions of hopelessness. This disclosure is often beneficial in the moment. However, it occurs within a commercial context, against terms of service that few users read and fewer fully understand, with data retention practices that may extend the intimacy of the conversation well beyond its duration. The consent framework that most AI chatbots use today is wordy and full of dense legalese, making it difficult for an average person to understand, much less agree to. If patients are not being genuinely informed to give consent, then they cannot be genuinely empowered to be participatory.

## AI as Romantic Partner

While the therapeutic and companionship uses of AI are straightforward, the growing use of romantic chatbots may be less understandable. The research base for this kind of AI use is also in its infancy, relying on observational data rather than the randomized controlled studies used to examine AI use in therapy. The study by Willoughby et al [25] of a US sample of young adults found that romantic engagement with AI is not rare. A meaningful subset of users engaged with AI chatbots specifically to replicate romantic experiences, and the behavior was not concentrated among any identifiable demographic group [25]. A mixed methods analysis by Wang et al [26] similarly documents the complexity and diversity of human-AI romantic relationships. People bring a wide range of motivations, attach a wide range of meanings, and describe both positive and negative outcomes, making simplistic explanations for the growing use of AI as a romantic partner impossible [26].

The psychological mechanisms underlying romantic AI attachment are similar to those in companionship but are intensified by the introduction of intimacy cues. A romantic chatbot can be consistently affirming, attentive to the user’s stated preferences, physically (textually) affectionate, and resistant to the conflicts, competing needs, and emotional ups and downs that can characterize human romantic relationships. Communication researchers have described parasocial relationships—one-sided emotional bonds with celebrities, media figures, or influencers—as a normal component of media consumption [27]. AI romance differs from classic parasocial attachment because the system responds, adapts, and appears to reciprocate a user’s thoughts and feelings. This responsiveness may create something closer to the felt experience of reciprocal intimacy than any prior media technology has generated. This can make it feel both more valuable and more consequential.

For individuals with social anxiety, a physical disability, or relational trauma or for those who live in geographic isolation, AI romantic interaction may provide concrete benefits. Romantic AI chatbots can provide emotional comfort, a sense of being desired and valued, an opportunity to practice communication and boundary setting, and a space to explore aspects of identity and preference that feel too vulnerable to risk in a human relationship. Attachment theory proposes that experiences of felt security—regardless of their source—contribute to the development of more secure internal working models of the self and others [28]. For individuals with histories of insecure or traumatic attachment, a consistent, nonthreatening, caring virtual relationship may have significant value.

However, the risks are more serious than in companionship use and may be harder to mitigate. The first risk concerns the calibration of expectations. An AI romantic partner engineered for engagement will, by design, be more attentive, patient, consistently affirming, and free of competing needs than any human partner can be. Users who develop an emotional attachment in this context may come to experience the ordinary friction of human romantic relationships—misattunements, competing needs, and imperfect availability—as aberrant rather than as a naturally occurring component of real-life relationships.

The second risk is dependency, resulting in real-life avoidance. Attachment theory suggests that avoidant relational strategies are maintained through the reinforcement they provide in the short term: avoided situations do not disconfirm the fears that motivate avoidance [28]. An AI romantic partner that is perfectly safe, consistently available, and immune to rejection may provide reinforcement for permanent avoidance. The chatbot may provide the affective benefits of attachment without requiring the vulnerability through which avoidant patterns are actually healed.

Romantic AI use has some of the greatest privacy concerns. The content of these interactions—expressions of desire, disclosures of vulnerability, and intimate exchanges—is among the most sensitive personal information that exists, generated within a commercial context that users rarely think about at the moment of disclosure. Data governance rules for romantic AI platforms are not merely good practice but are required and need to be understood by users. Finally, the hallucination and sycophancy risks that run through all AI mental health applications take on particular weight in a romantic context. A system that affirms a user’s most distorted beliefs about themselves, their ex-partners, or their worthiness of love is not merely providing inaccurate information—it is doing so through a medium specifically designed to maximize trust and emotional receptivity [5,6].

## Cross-Cutting Mechanisms and Safety Challenges

Across all 3 of these roles, a set of underlying mechanisms accounts for both the benefits and challenges that accompany AI mental health chatbots.

The most significant mechanism is the one Reeves and Nass [3] identified before LLMs existed: the social brain does not have a reliable switch for distinguishing human from nonhuman interactions. When a system produces socially cued outputs—responsive turn taking, emotionally attuned language, and apparent interest—the mind processes it socially, applies interpersonal norms, and generates affective responses appropriate to a social or relational encounter. Conversational AI has intensified this tendency to a degree that its predecessors could not achieve. The result is not user error or technological naivety but rather the activation of cognitive and emotional systems that evolved long before the distinction between person and machine had any relevance.

A closely related mechanism is the online disinhibition effect [29]. Suler [29] identified 6 factors that collectively loosen the behavioral constraints governing self-presentation and disclosure: dissociative anonymity, which severs the link between online behavior and offline identity; invisibility, which removes the audience cues that trigger social monitoring; asynchronicity, which attenuates the sense of real-time social consequence; solipsistic introjection, which leads people to experience the interaction as self-generated rather than truly witnessed; dissociative imagination, which frames the interaction as consequence free; and minimization of authority, which removes the power differentials that ordinarily regulate disclosure. In AI relational contexts, several of these factors operate simultaneously and with unusual intensity. The user is not visible to any social audience. The AI carries no institutional authority and imposes no evaluative judgment. The interaction can feel like a kind of projective space—responsive enough to seem real but, ultimately, of the user’s own creation. This combination is what makes AI disclosure so easy. The same conditions that make it easier to say difficult things also reduce the normal safeguards on what we say, to whom we say it, and at what cost.

Recent empirical work by Syrjämäki et al [30] illustrates the challenges presented by the online disinhibition effect on behavior. Their analysis demonstrates that online disinhibition mediates the relationship between emotion regulation difficulties and harmful communication behavior. The researchers found that individuals who struggle to regulate emotional arousal become more disinhibited online and this disinhibition predicts impulsive, boundary-crossing behavior.

The implications for AI mental health chatbots are noteworthy. The same reduction in felt social consequence that makes it easier to disclose trauma to an AI also makes it easier to spiral into escalating emotional expression, share information one later regrets, or pursue relational dynamics one’s own emotional regulation would normally prevent in a human context. Disinhibition requires careful attention and direction, not maximization. Designing a mental health chatbot’s beneficial expressions while limiting its harmful ones is one of the central challenges of responsible AI design.

Two additional mechanisms operate at the level of the AI system. The first is engagement optimization via sycophancy. General commercial AI systems are typically trained to prioritize user satisfaction and continued engagement. These are metrics more readily produced via agreement, validation, and affirmation than via challenge, disruption, or the friction sometimes generated by honest conversations. The sycophancy literature documents the result: systematic tilting toward whatever position the user appears to hold, reinforcement of confidently expressed beliefs, and suppression of the productive disagreement that therapeutic relationships (and many strong friendships) depend on [6]. This is a structural consequence of optimizing for engagement. Addressing it requires a deliberate design commitment to prioritize therapeutic fidelity over user-reported satisfaction, which in turn requires evaluation frameworks that can distinguish between the two.

For shared decision-making to be effective in participatory medicine, interactions must be honest and based on facts as well as clean data. When these data become polluted through sycophantic bias, the resulting conversation will be less than honest. This is a hidden challenge in using AI for participatory medicine because informed self-care comes not only from data and knowledge about a health condition but also from accurate and sometimes challenging conversations about treatment options and likely outcomes. When AI is optimized for engagement rather than honesty and challenging the user’s maladaptive thoughts, feelings, or behaviors, it becomes more of an ineffective but friendly echo chamber and less of a useful health consultant.

The second mechanism is hallucination—the capacity of generative systems to produce fluent, confident, and completely fabricated content with no internal signal flagging the fabrication [5]. In casual information-seeking contexts, hallucination is an inconvenience. In mental health contexts, where users may be making decisions about medication, safety, diagnosis, and treatment, this is a patient safety issue. Language models produce the most statistically probable continuation of a prompt, which may or may not correspond to anything true in the world. There is no known technical fix at this time that resolves this concern. The practical implication for mental health AI is that users must be genuinely informed about AI’s limitations (not just in the fine print or terms of service). Any kind of clinical guidance from an AI chatbot should be understood as provisional rather than authoritative.

Together, these mechanisms suggest a more demanding conception of safety than the industry typically adopts. Adding a crisis hotline number at the bottom of every chatbot conversation is not a safety feature; it is simply a liability hedge. Real safety requires designing systems that are honest about what they are, actively promote human connection, detect clinical risk with enough sensitivity and specificity to be trusted, and treat the user’s long-term well-being and need for human relationships as design guardrails rather than secondary considerations [31].

## Implications for Participatory Medicine

Participatory medicine is sometimes understood as a delivery philosophy—getting patients more involved, giving them better tools, and making decision-making a more equal and two-way process. However, at its core, it is a set of commitments about what good health care actually is: not the transfer of expertise from clinician to patient but the partnership of two people committed to understanding, care, and health within a relationship that takes both parties equally seriously. Conversational AI is relevant to this vision in ways that are both promising and potentially challenging. Participatory medicine has the potential to help inform the conversation.

The promising side begins with access. For most people who have mental health needs but cannot access treatment—due to cost, geography, wait times [11], stigma [13], or scheduling availability—an AI system can provide hope. Mental health AI chatbots can offer an invaluable bridge to people in need through structured exercises, reflection, psychoeducation, and the felt experience of being heard. AI tools examined in the literature have produced real symptom reduction [15,16]. Between-session support, help in articulating concerns for upcoming appointments, and guidance through evidence-based CBT exercises are genuine contributions to care, particularly for individuals navigating the system for the first time [32]. AI systems can also help with initial engagement with mental health care, reaching people before they have found a clinician and supporting them in ways that make the eventual clinical encounter more productive.

The challenging side is more subtle but equally urgent. AI systems that optimize for engagement will, absent deliberate countermeasures, tend to become what users *want* them to be rather than what users *need* them to be. They will validate a person’s thoughts or feelings when they should challenge them instead, agree when they should question, and sustain conversations that a responsible clinician would redirect. The felt experience of being understood by an AI chatbot that does not actually understand, reinforced over time, may substitute the human understanding that participatory medicine identifies as core to genuine patient engagement. The risk is quiet, gradual, and easy to rationalize. The user reports feeling better; the engagement metrics on the surface appear to be strong; and the harm is almost impossible to attribute to any particular, single interaction.

Several implications follow for design, practice, and policy. First, transparency must be understood not as a disclosure obligation but as a clinical feature. Users of AI mental health systems should come away from every interaction with an accurate understanding of what the system is and is not capable of, how its outputs are generated, and how their data are used. This is not accomplished by a terms-of-service agreement or a one-time onboarding screen. Instead, it requires ongoing, contextually appropriate communication embedded in the interaction itself.

Second, escalation pathways must be designed with the same care as the conversational interface. The gap between recognizing a crisis and reaching human support is where the most preventable harms occur. Closing this gap requires active design interventions rather than passive disclaimers. AI systems should be designed to detect clinical risk, reduce friction in accessing help, and maintain a connection with the user throughout the transition.

Third, the evaluation framework for AI mental health tools must expand beyond symptom outcomes and satisfaction ratings to include relational outcomes—what happens to users’ human relationships over time; whether AI use functions as a bridge or as a retreat; and whether the capacity for human intimacy is preserved, strengthened, or eroded [24]. This may be methodologically demanding and commercially inconvenient, but it is important to address when AI is being used for mental health.

Fourth, clinicians need preparation. Patients who describe their AI companion as a central source of support or their AI romantic partner as more emotionally available than any human relationship they have are already in clinical practice—and they deserve responses that are neither dismissive nor uncritically validating. Participatory medicine frameworks that prepare clinicians for these conversations without stigmatizing what patients find meaningful could be significantly helpful [26].

Finally, the pace of adoption has outrun the pace of evidence. The most vulnerable users are already inside systems that have not been adequately evaluated, and the normative frameworks for what adequate evaluation even looks like are still being developed. Participatory medicine can contribute something important: a commitment to centering the lived experience of users—including their experiences of harm that never make it into randomized trials—as evidence that counts and a governance approach that treats the communities most affected by these technologies as partners in their evaluation rather than subjects of it [17].

## Conclusions

Conversational AI can provide benefits in some relational contexts for some users under conditions we are only beginning to understand. These same features that make it beneficial also make it capable of causing harm that is often difficult to understand and appreciate until it has accumulated over time.

The 3 relational roles examined here—therapist, companion, and romantic partner—are not merely categories for organizing the literature. They are windows into different dimensions of human need: the need for guidance through distress, for connection in isolation, and for intimacy in its most vulnerable form. AI can help in each of these areas, filling gaps that human care systems have failed to close [9,11,12]. This is more a factor of the vast scale of unmet need for mental health treatment options as much as it is about this emerging technology.

The path forward is not a choice between embracing AI and protecting human connection but, rather, designing systems, evaluation frameworks, clinical practices, and governance structures that do both. Conversational AI should be honest about its nature and limitations and built to strengthen the conditions for human care rather than substitute it; it should be judged on its long-term effects on users’ lives, not just their mood in the hour after an interaction; and it should be developed with the active participation of the people whose vulnerability it most directly engages. Participatory medicine is not a by-product of good AI design; it is what makes that design possible—patients engaging as equals in their own care.

## Abbreviations

CBT: cognitive behavioral therapy
LLM: large language model

## Data Availability

This Viewpoint synthesizes previously published empirical literature and does not report original research data. No new datasets were generated or analyzed. All source articles are cited and publicly available through their respective publishers.

## Editorial Notice

The author cofounded the Society for Participatory Medicine. The editor serves as editor-in-chief of the *Journal of Participatory Medicine*, the official journal of the Society for Participatory Medicine, and serves in an ex officio position on the Society for Participatory Medicine board of directors.
